# Mechanisms of angioregression of the corpus luteum

**DOI:** 10.3389/fphys.2023.1254943

**Published:** 2023-09-28

**Authors:** Corrine F. Monaco, John S. Davis

**Affiliations:** ^1^ Department of Cellular and Integrative Physiology, University of Nebraska Medical Center, Omaha, NE, United States; ^2^ Department of Obstetrics and Gynecology, University of Nebraska Medical Center, Omaha, NE, United States; ^3^ US Department of Veterans Affairs Nebraska-Western Iowa Healthcare System, Omaha, NE, United States

**Keywords:** corpus luteum, angioregression, endothelial cells, PGF2α, luteolysis, ovary

## Abstract

The corpus luteum is a transient ovarian endocrine gland that produces the progesterone necessary for the establishment and maintenance of pregnancy. The formation and function of this gland involves angiogenesis, establishing the tissue with a robust blood flow and vast microvasculature required to support production of progesterone. Every steroidogenic cell within the corpus luteum is in direct contact with a capillary, and disruption of angiogenesis impairs luteal development and function. At the end of a reproductive cycle, the corpus luteum ceases progesterone production and undergoes rapid structural regression into a nonfunctional corpus albicans in a process initiated and exacerbated by the luteolysin prostaglandin F2α (PGF2α). Structural regression is accompanied by complete regression of the luteal microvasculature in which endothelial cells die and are sloughed off into capillaries and lymphatic vessels. During luteal regression, changes in nitric oxide transiently increase blood flow, followed by a reduction in blood flow and progesterone secretion. Early luteal regression is marked by an increased production of cytokines and chemokines and influx of immune cells. Microvascular endothelial cells are sensitive to released factors during luteolysis, including thrombospondin, endothelin, and cytokines like tumor necrosis factor alpha (TNF) and transforming growth factor β 1 (TGFB1). Although PGF2α is known to be a vasoconstrictor, endothelial cells do not express receptors for PGF2α, therefore it is believed that the angioregression occurring during luteolysis is mediated by factors downstream of PGF2α signaling. Yet, the exact mechanisms responsible for angioregression in the corpus luteum remain unknown. This review describes the current knowledge on angioregression of the corpus luteum and the roles of vasoactive factors released during luteolysis on luteal vasculature and endothelial cells of the microvasculature.

## 1 Introduction

Vascular remodeling occurs in various disease states such as myocardial infarction, traumatic spinal cord injury (Olive et al., 2003; Benton et al., 2008), neurovascular disease (Planas, 2020), and various hypertensive disorders (Intengan and Schiffrin, 2001). Degradation of blood vessels, or angioregression, also occurs in disease states such as chronic graft nephropathy (Ishii et al., 2005) and muscle degeneration (Dedkov et al., 2002; Malek et al., 2010; Olenich et al., 2014). Vast non-pathological angioregression occurs during normal development (Yin and Pacifici, 2001; McKeller et al., 2002; Nayak et al., 2018); however, it is not prevalent in most adult tissues. There are some instances where physiological angioregression occurs, such as in uterine remodeling during pregnancy (Henry et al., 2006), mammary gland involution (Djonov et al., 2001), and during luteolysis of the ovarian corpus luteum during the female reproductive cycle (Augustin, 2000).

The corpus luteum is a temporary, dynamic endocrine gland formed from the remnants of the ovulated follicle (Stocco et al., 2007). It produces the progesterone that is necessary for successful establishment and maintenance of pregnancy. Following ovulation, the remaining granulosa and theca cells of the ovulated follicle undergo rapid, but limited, division and then differentiation as the ovary shifts from making estradiol to making progesterone in a process known as luteinization (Smith et al., 1994; Fraser and Duncan, 2009). Notably, endothelial cells from the theca layer of the follicle undergo rapid division, comparable to that of tumor angiogenesis, forming the vast microvasculature of the corpus luteum, with around 50% of cells in the mature corpus luteum comprised of endothelial cells (Rodgers et al., 1984; O’shea et al., 1989). In addition to the steroidogenic and endothelial cells, the corpus luteum contains small populations of fibroblasts, mesenchymal-like cells that produce and regulate extracellular matrix, and immune cells (O’shea et al., 1989; Van Linthout et al., 2014). The luteal vasculature contains mostly capillaries, and nearly every steroidogenic cell is in contact with a microvascular endothelial cell (Zheng et al., 1993; Fraser and Duncan, 2009). As a result, the corpus luteum has one of the highest blood supplies, per unit of any organ system (Smith et al., 1994), as well as high blood flow and low vascular resistance compared to the surrounding ovarian stroma (Wiltbank et al., 1990). The microvasculature is crucial for luteal function, as blockage of angiogenesis during luteal formation results in dysfunctional corpora lutea (Yamashita et al., 2008). The luteal vasculature is important for both transport of progesterone from steroidogenic cells to the rest of the body and for nourishment of the corpus luteum (Woad and Robinson, 2016). Early studies in rabbits demonstrated the importance of the corpus luteum for the production of progesterone and pregnancy (Mesiano, 2022). In women, luteal function is crucial for pregnancy, as removal of the corpus luteum within the first 7 weeks of pregnancy results in termination of said pregnancy (Csapo et al., 1974). Progesterone secreted from the corpus luteum is crucial for creating optimal conditions for embryo implantation, a crucial point in pregnancy (Kolatorova et al., 2022).

In a reproductive cycle that does not result in pregnancy, the corpus luteum must cease progesterone production so the next reproductive cycle can begin. In many species including bovines (Knickerbocker et al., 1988), ovines (Goding, 1974), guinea pigs (Evans, 1987), and rats (Wang et al., 1993), luteal regression (luteolysis) is initiated by pulses of prostaglandin F2α (PGF2α) produced by the uterus. In primates, it is believed to be initiated by the lack of gonadotropin support and a concomitant rise in intra-ovarian PGF2α production (Stouffer et al., 2013). Luteolysis occurs in two steps: (1) functional regression, in which progesterone production declines, and (2) structural regression, in which the corpus luteum dissipates into a fibrotic corpus albicans within the ovarian stroma. Structural regression involves various pathways of tissue remodeling, including immune cell infiltration, breakdown and buildup of extracellular matrix, and regression of the microvasculature. Although it is known that blood flow is impaired and the vascular endothelial cells are sensitive to various factors released during luteolysis, the exact mechanism of angioregression in the corpus luteum remains unknown. This review will highlight current knowledge on angioregression of the corpus luteum: from macrovascular changes, dilation and constriction of luteal vasculature and subsequent impedance of blood flow, to microvascular changes, such as the effects of growth factors, cytokines, and thrombospondins on microvascular endothelial cells.

## 2 Blood flow

**FIGURE 1:**
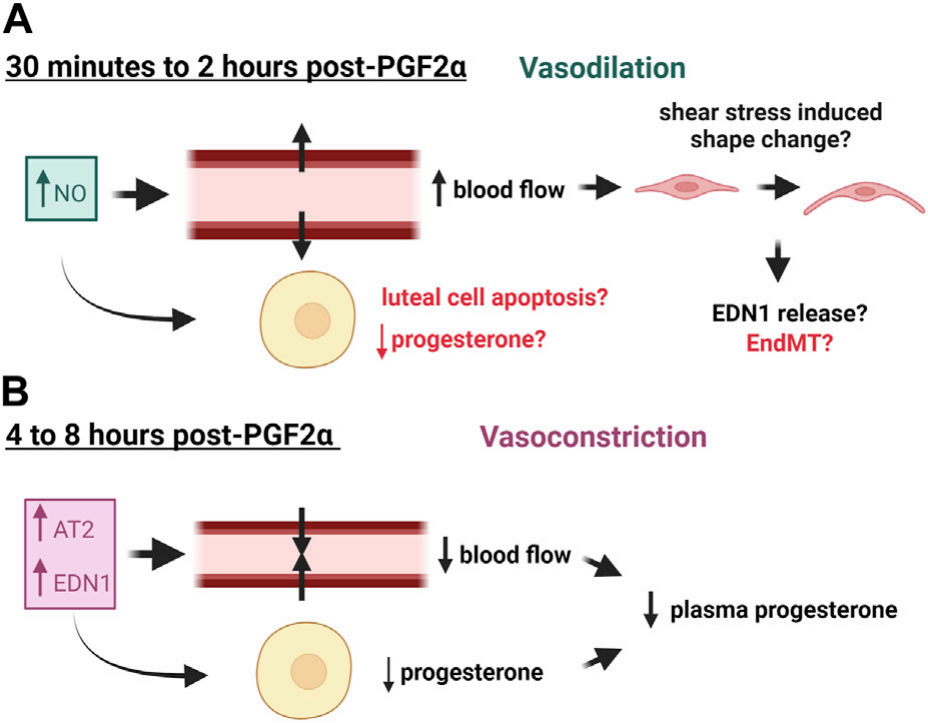
Effects of Vasoactive Factors released in response to PGF2α on blood flow of the corpus luteum. **(A)** Nitric Oxide (NO) is released early in luteal regression and causes vasodilation and subsequent increased blood flow, which may consequently alter endothelial cell phenotype. **(B)** Later in luteal regression, vasoconstrictors such as Angiotensin II (AT2) and endothelin 1 (EDN1) are released, causing decreased blood flow and decreased plasma progesterone. These factors may also impair progesterone production of luteal steroidogenic cells. This figure was made with Biorender.com.

Electron microscopy of regressing guinea pig and bovine corpora lutea revealed that endothelial cells detach from the basement membrane, slough off the capillaries, and clog the vessels; yet the capillary walls remain intact (Azmi and O'Shea, 1984; Modlich et al., 1996). However, luteal capillaries eventually disappear throughout regression. Twelve hours after PGF2α-induced regression, there were noticeably fewer capillaries in ovine corpora lutea (Nett et al., 1976), but more arterial vessels were present (Bauer et al., 2003). These vessels contain thicker walls of smooth muscle (Bauer et al., 2003), which is further evidenced by increased smooth muscle actin positive cells surrounding arterioles in regressing bovine corpora lutea (Hojo et al., 2009). The diameter of these vessels was demonstrated to be smaller compared to fully functional mid-cycle corpora lutea (Lei et al., 1991; Nio-Kobayashi et al., 2016), indicating that vasoconstriction may be occurring in luteal regression. Similarly, arterial wall thickening is associated with various cardiovascular diseases including atherosclerosis, thrombosis, and hypertension (Burke et al., 1995; Corinaldesi and Corinaldesi, 2008). Changes in blood flow during luteal regression as a result of vasoactive factors are illustrated in Figure 1.

Early studies in the rabbit indicate that luteal blood flow is highly correlated to progesterone levels in pseudopregnant rabbit corpora lutea (Janson et al., 1981). Due to their size and prominent vascular blood supply, corpora lutea from larger species such as ruminants and humans can be identified via ultrasonography. Doppler ultrasound has been utilized to study corpora lutea during natural and PGF2α-induced regression. Doppler readings in human corpora lutea have identified that blood flow drops in the days nearing menstruation, which correlates to the reduction in progesterone production in the late luteal phase (Miyazaki et al., 1998), and pregnant cattle exhibited a much higher luteal blood flow than non-pregnant cattle (Kanazawa et al., 2022). Additionally, *in vivo* studies of cows injected with PGF2α to induce luteolysis have identified that there is an early increase in blood flow after 2 h of treatment that ultimately decreases below baseline levels after 8 h of treatment (Acosta et al., 2002; Miyamoto et al., 2005; Jonczyk et al., 2021). A study in the ovine suggested that ovarian blood flow to the luteal ovary decreased 4 h post-PGF2α treatment, followed by a decline in progesterone 6 h post-PGF2α treatment (Nett et al., 1976). Similar findings were reported in the equine and the donkey (Ginther et al., 2007; Miró et al., 2015). Also, luteal blood flow and plasma progesterone followed a more similar trend in the cycling bovine corpus luteum than did luteal size, which is commonly used to identify luteal stage (Herzog et al., 2010), thus implicating blood flow as an accurate measure of luteal function. However, the opposite has been determined for the midcycle in lactating dairy cows (Lüttgenau et al., 2011). The reason for the discrepancy is likely due to the enhanced hepatic metabolism of progesterone in lactating dairy cows (Wiltbank et al., 2006).

The transient increase in blood flow seen early in luteal regression is believed to be a result of the increased synthesis of the vasodilator, nitric oxide (NO) (Miyamoto et al., 2005; Shirasuna et al., 2008). Evidence across various species has shown that shear stress from increased blood flow changes the shape of vascular endothelial cells from their signature cobblestone shape to one that is more spindle-like and oriented towards the direction of flow (reviewed in (Masuda et al., 2003; Campinho et al., 2020)). This has been observed in endothelial cells derived from the carotid arteries of rabbits (Masuda et al., 1999) and mouse cardiac and pulmonary endothelial cells (Merna et al., 2018). High stretch of cultured endothelial cells induces a similar phenotype in which the cells acquire a spindle-like shape via phalloidin remodeling and produce increased reactive oxygen species (Girão-Silva et al., 2021). This change in endothelial cell phenotype is suggested to be mediated by calcium signaling and tyrosine kinase phosphorylation in cultured bovine aortic endothelial cells (Malek and Izumo, 1996). More recent studies show that increased shear stress causes retinal endothelial cells cultured in 3D (Luu et al., 2023) and human aortic endothelial cells (Kidder et al., 2023) to adopt a more mesenchymal phenotype. There are no reports to our knowledge of this phenomenon occurring in luteal endothelial cells; however, shear stress from a compensatory blood flow increase early in luteal regression may contribute to the changes in capillary basement membrane that are seen during regression, which could result in the release of endothelial cells from the capillary. Another hypothesis is that the shear stress could cause the endothelial cells to transition to a mesenchymal phenotype, as increased matrix stiffness is associated with fibrosis and endothelial cell destabilization (Yu et al., 2023). It is also important to note that endothelial cells are sensitive to many factors released during luteal regression as discussed later; therefore, changes in blood flow are likely not the sole contributor to luteal angioregression.

## 3 Effect of vasoactive factors on luteal vasculature and endothelial cells

Outside of the corpus luteum, PGF2α is more commonly known as a vasoconstrictor, in which it does so by activating calcium signaling and inducing contraction of smooth muscle cells (Watts, 2007). In addition to PGF2α, various other vasoactive agents are increased in luteal regression, such as NO, angiotensin II (AT2), and endothelin 1 (EDN1) (Davis et al., 2003; Skarzynski et al., 2008; Shirasuna et al., 2012b). The known changes in vasoactive factors contributing to blood flow in luteal regression are summarized in Table 1.

**TABLE 1:** Summary of Vasoactive, Pro-angiogenic, and Anti-angiogenic factors that are regulated during luteal angioregression.

	Factor	Expression during luteal regression	Source	Effect on endothelial cells	Proposed role in angioregression	References
Vasoactive factors						
NO	Nitric Oxide	↑ (early)	Endothelial cells (eNOS)	Inhibits apoptosis	Vasodilation, increased blood flow, stimulation of PGF2α	(Dimmeler and Zeiher, 1999; Motta et al., 1999; Fridén et al., 2000; Tao et al., 2004; Shirasuna et al., 2008; Förstermann and Sessa, 2012)
			Steroidogenic cells (iNOS)			
AT2	Angiotensin II	↑	Circulating, ovary	Proliferation	Vasoconstriction, increase PGF2α	(Hayashi and Miyamoto, 1999; Benigni et al., 2010)
EDN 1	Endothelin 1	↑	Endothelial cells	Proliferation, prostaglandin release	Vasoconstriction	(Girsh et al., 1996b; Hinckley and Milvae, 2001; Tanfin et al., 2011)
Pro-angiogenic factors						
VEGF	Vascular Endothelial Growth Factor	↓	Perivascular cells, steroidogenic cells	Proliferation	Decrease during luteal regression causes vessel dysfunction	(Gospodarowicz et al., 1989; Hazzard et al., 2000; Neuvians et al., 2004a)
FGF2	Fibroblast Growth Factor 2	↑	Steroidogenic cells and endothelial cells	Proliferation	Fibroblast division, sensitization of endothelial cells (via increased type 1 collagen)	(Gospodarowicz, 1974; Asakai et al., 1993; Robinson et al., 2007; Maroni and Davis, 2011; Zalman et al., 2012; Alan and Kulak, 2021; Monaco et al., 2023)
ANGPT1	Angiopoietin 1	__	Perivascular cells, steroidogenic cells	Proliferation	Decrease in relation to ANGPT2 inhibits angiogenesis	(Maisonpierre et al., 1997; Wulff et al., 2000; Sugino et al., 2005; Fiedler et al., 2006; Eklund and Saharinen, 2013)
Anti-angiogenic factors						
ANGPT2	Angiopoietin 2	↑	Endothelial cells, potentially steroidogenic cells	Inhibits ANGPT1-induced effects (Tie2 antagonist)	Inhibits new angiogenesis, sensitization of endothelial cells	(Maisonpierre et al., 1997; Wulff et al., 2000; Tanaka et al., 2004; Sugino et al., 2005; Eklund and Saharinen, 2013)
THBS1	Thrombospondin 1	↑	Endothelial cells, steroidogenic cells	Apoptosis (via TGFB1)	Endothelial cell death, vessel fibrosis (synergy with TGFB1)	(Mondal et al., 2011; Zalman et al., 2012; Berisha et al., 2016b; Farberov and Meidan, 2016)
TNF	Tumor Necrosis Factor α	↑	Immune cells, endothelial cells, possibly steroidogenic cells	Apoptosis	Endothelial cell death, PGF2α production	(Sakumoto et al., 2000; Pru et al., 2003; Cherry et al., 2008; Henkes et al., 2008; Sakumoto et al., 2011; Talbott et al., 2017)
IL1B	Interleukin 1β	↑	Immune cells (CD11b^+^), potentially fibroblasts and steroidogenic cells	Chemokine release	Immune cell recruitment, PGF2α production, potentially vessel fibrosis via EndMT	(Kobayashi and Terao, 1997; Estevez et al., 2003; Lopez-Castejon and Brough, 2011; Bourdiec et al., 2013; Bishop et al., 2017; Talbott et al., 2017; Baddela et al., 2018)
TGFB1	Transforming Growth Factor β1	↑	Ubiquitous	Apoptosis, dysregulation of capillary structures	Endothelial cell death, vessel fibrosis	(Branton and Kopp, 1999; Maroni and Davis, 2011; Maroni and Davis, 2012; Talbott et al., 2017)

### 3.1 Nitric oxide (NO)

Nitric oxide (NO) is commonly known as a vasodilator that is produced by nitric oxide synthase (NOS) enzymes. There are three identified NOS enzymes (NOS1-3), two of the three being uniquely expressed in specific cell types, neuronal NOS (nNOS or NOS1) and endothelial NOS (eNOS or NOS3). The third NOS enzyme is inducible NOS (iNOS or NOS2) (Förstermann and Sessa, 2012). Both eNOS and iNOS have been identified in luteal tissue, with eNOS primarily being localized to the endothelial cells and iNOS exhibiting weak localization to the steroidogenic cells (Fridén et al., 2000; Tao et al., 2004). One study identified increased eNOS in naturally regressing bovine corpora and in corpora lutea induced to regress following treatment with PGF2α (Shirasuna et al., 2008). Studies in the human report increased iNOS at mid and late luteal phase (Vega et al., 2000). However, others reported decreased eNOS immunolocalization and transcription during the bovine late luteal phase and luteal regression (Rosiansky-Sultan et al., 2006). Such differences can be attributed to differences in staging of the corpus luteum as opposite effects of PGF2α treatment were seen in early corpora lutea compared to midcycle corpora lutea, as well as whether the tissue sample was in the middle or periphery of the corpus luteum, as eNOS was elevated only in the periphery of midcycle corpora lutea in the bovine (Shirasuna et al., 2008). Direct intra-luteal delivery of an NO donor temporarily increased blood flow but then caused both blood flow and progesterone to fall to levels below baseline (Shirasuna et al., 2008). Similar results have been reported in other species. For instance, NO donor treatment decreased serum progesterone but increased ovarian PGF2α production in pseudopregnant rats, and the same has been reported in cultured human luteal cells (Motta et al., 1999; Fridén et al., 2000). Such differences may be due to pharmacologic administration of NO donors *in vivo* and species-to-species variations.

In addition to its ability to stimulate PGF2α, it has been suggested that NO may have a direct effect on luteal cells. L-arginine, an NOS substrate, increased apoptosis of cultured human luteal tissue, which was suppressed by treatment with an NO inhibitor (Vega et al., 2000). Similarly, treatment of bovine luteal cells with the NO donor, NONOate, increased levels of cleaved caspase 3, a marker of apoptosis (Korzekwa et al., 2006; Kowalczyk-Zieba et al., 2014). Conversely, treatment of luteinized goat granulosa cells with NO donor increased progesterone production, whereas treatment with an NOS inhibitor decreased progesterone and increased apoptosis (Guo et al., 2019). Such differences may be due to differences in culture conditions and the presence of multiple cell types (endothelial cells and immune cells) in the cultures. Collectively, these data indicate that NO may contribute to luteolysis beyond the vasculature and may have a direct effect on steroidogenesis. Although there is some evidence that NO may be an inhibitor of apoptosis in endothelial cells (Dimmeler and Zeiher, 1999, this phenomenon has yet to be studied in luteal endothelial cells.

### 3.2 Angiotensin II (AT2)

Angiotensin II (AT2) is a potent vasoconstrictor that is derived from angiotensinogen produced by the liver. Angiotensinogen is converted to angiotensin I by the enzyme renin, and angiotensin I is converted to angiotensin II by angiotensin converting enzyme (ACE) (Ng and Vane, 1967). AT2 has pleiotropic effects that include production of aldosterone, which increases blood pressure, or it can act as an independent vasoactive factor or act on other systems such as the kidney to raise blood pressure (Fyhrquist et al., 1995; Santos, 2014). Such effects, along with pro-inflammatory and pro-fibrotic effects are mediated by the angiotensin receptor 1 (AGTR1) (Benigni et al., 2010). Angiotensin II is also capable of binding to the AGTR2, which induces vasodilation and anti-inflammatory and anti-fibrotic effects (Benigni et al., 2010). Interestingly, AGTR1 levels remain consistent throughout the luteal phase, but AGTR2 levels are decreased at midcycle (days 8–12) but are increased during the late luteal phase (days 13–17) and regression (> day 18) in bovine corpora lutea, but were also found to be elevated to the same level in the corpus luteum of pregnancy (Hayashi et al., 2000; Kobayashi et al., 2001; Schams et al., 2003). Although this seems contradictory to the increased inflammation and fibrosis seen during luteal regression, it is possible that (1) these studies only accounted for AGTR2 mRNA and not for desensitization or endocytosis of the AGTR2, or (2) AGTR2 actions ensure that the regression is tightly controlled.

The ovary is capable of producing renin and AT2 (Yoshimura, 1997; Palumbo et al., 2016). Additionally, ACE has been identified in endothelial cells of the bovine corpus luteum during early luteal phase (Hayashi et al., 2000). The luteal renin-angiotensin-aldosterone system (RAAS) may serve an important role in pregnancy in women, as increased corpus luteum number was associated with differential levels of RAAS components, but there were no differences in birth outcomes (Wiegel et al., 2021).

PGF2α has been shown to increase AT2 levels in microdialyzed bovine corpora lutea *in vitro* (Hayashi and Miyamoto, 1999), and AT2 has been demonstrated to increase PGF2α levels but also progesterone levels alone and in conjunction with PGF2α treatment *in vitro* (Kobayashi et al., 2001). In that study, AT2 increased secretion of oxytocin, another luteolytic factor. In rat luteal cells, AT2 had no effect on basal progesterone, but inhibition of AGTR2 slightly decreased progesterone secretion (Pepperell et al., 2006). However, in another study, AT2 decreased progesterone after 14 h alone and after 4 h in conjunction with PGF2α in microdialyzed bovine corpora lutea (Hayashi and Miyamoto, 1999). It is possible that such effects are mediated by angiotensin reactive proteins in the luteal endothelial cells, as ACE is present in bovine luteal endothelial cells, and treatment with an ACE inhibitor, captopril, decreased AT2 secretion from luteal endothelial cells (Hayashi et al., 2000). Therefore, it is possible that endothelial-derived AT2 may aid in decreasing progesterone either alone or through the release of luteolytic factors.

### 3.3 Endothelin-1 (EDN1)

Endothelin 1 (EDN1) is a 21 amino acid protein derived from endothelial cells that is known to be a potent vasoconstrictor (Yanagisawa et al., 1988). It is produced in endothelial cells as a larger pro-endothelin which is then cleaved to active endothelin by endothelin converting enzyme (ECE) (Sawamura et al., 1989; Tanfin et al., 2011). Studies in the cow and the ewe have identified that luteal endothelin 1 levels increase during natural and PGF2α-induced luteal regression (Girsh et al., 1996b; Ohtani et al., 1998; Hinckley and Milvae, 2001), and PGF2α induces secretion of EDN1 from microdialysed bovine corpora lutea (Miyamoto et al., 1997). Furthermore, tumor necrosis factor α (TNF), an inflammatory cytokine that is elevated in luteal regression, increased EDN1 production by bovine aorta endothelial cells (Marsden and Brenner, 1992) therefore EDN1 secretion may be regulated by PGF2α, inflammatory cytokines, and other secondary responses to PGF2α.

Although EDN1 is well known for its role as a vasoconstrictor, it may have some direct effects on steroidogenic cells in the corpus luteum. *In vitro,* EDN1 decreased progesterone secretion of ovine luteal minces and decreased basal and LH-induced progesterone production by large, but not small bovine luteal cells (Girsh et al., 1996a; Doerr et al., 2008). *In vivo*, EDN1 decreased progesterone in a synergistic manner with PGF2α in the bovine (Shirasuna et al., 2006). Given this information, the luteolytic role of EDN1 may involve both vasoconstriction and direct action on luteal steroidogenic cells.

## 4 Pro- and anti-angiogenic factors in luteolysis

**FIGURE 2:**
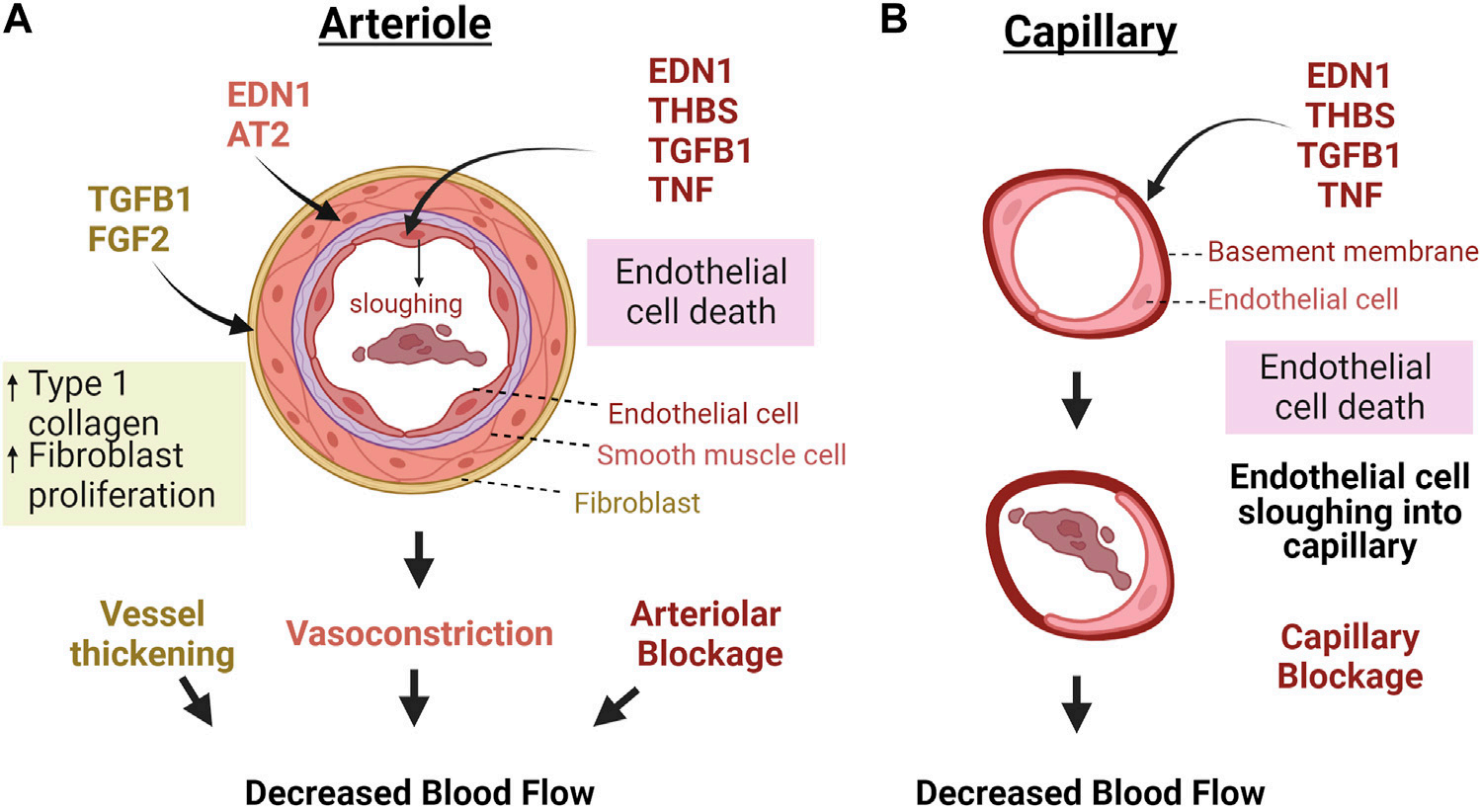
Macro vs. microvascular changes in response to factors secreted during luteal regression. **(A)** Because the luteal microvasculature also contains smooth muscle and pericytes, vasoactive factors cause contraction of vascular smooth muscle cells and consequent vasoconstriction, in addition to TGFB1 and FGF2 activating fibroblasts to produce more collagen, thereby thickening and stiffening the vessels. Capillaries do not have these bordering cells **(B)**. Therefore, these vessels would mostly be impacted by factors that directly impact endothelial cells. In both cases, death of endothelial cells causes sloughing into the capillaries which can clog vessels and impair blood flow. Abbreviations: Endothelin 1 = EDN1, Angiotensin II = AT2, Fibroblast growth factor 2 = FGF2, Transforming growth factor β-1 = TGFB1, Thrombospondin 1 = THBS1, Tumor necrosis factor α = TNF. The figure was made using Biorender.com.

In addition to the presence of factors affecting vessel dilation or contraction during luteal regression, luteolysis is associated with decreased expression of pro-angiogenic factors such as vascular endothelial growth factor (VEGF) and increased expression of anti-angiogenic factors, such as thrombospondin 1 (THBS1), EDN1, transforming growth factor β 1 (TGFB1) and pro-inflammatory cytokines (Skarzynski et al., 2008; Shirasuna et al., 2012b; Berisha et al., 2016a; Talbott et al., 2017). The proposed effects of these factors on luteal vasculature are illustrated in Figure 2. These events are triggered by a luteolytic dose of PGF2α *in vivo*; however, multiple studies have demonstrated that endothelial cells do not express the receptor for PGF2α (Liptak et al., 2005; Maroni and Davis, 2011). Therefore, the effects of the luteolytic cascade on microvascular endothelial cells must be from release of factors following the initial PGF2α pulses. Furthermore, luteal endothelial cells are known to be sensitive to such factors, and thus are believed to be the first to die during luteal regression (Azmi and O'Shea, 1984). Additionally, the luteal rescue signal, interferon tau, increases endothelial cell survival and decreases transcription of luteolytic factors like *THBS1*, *EDN1*, and *TGFB1* in bovine luteal slices (Basavaraja et al., 2017). Therefore, the presence or absence of secreted factors relating to angiogenesis may contribute to luteal angioregression.

### 4.1 Pro-angiogenic factors

#### 4.1.1 Vascular endothelial growth factor (VEGF)

Vascular endothelial growth factor (VEGF) is growth factor that acts as a potent mitogen on endothelial cells (Gospodarowicz et al., 1989). There are five VEGF isoforms which can bind to three receptors, VEGFR1 (Flt1), VEGFR2 (KDR or Flk1) and VEGFR3 (Flk4) (Geva and Jaffe, 2000). Because rapid angiogenesis is necessary to build the luteal microvasculature, VEGF is highly prominent in the developing corpus luteum (Redmer et al., 1996; Woad and Robinson, 2016). Interruption of VEGF during ovulation results in decreased luteal formation (Duncan et al., 2008). In the late luteal phase, and in PGF2α-induced regression across multiple species, luteal *VEGF* mRNA decreases (Otani et al., 1999; Hazzard et al., 2000; Neuvians et al., 2004a; Vonnahme et al., 2006). The rapid reduction in VEGF expression indicates that no active angiogenesis is occurring during luteal regression, a finding confirmed by studies across various species, including the rat (Tamura and Greenwald, 1987), sheep (Reynolds and Redmer, 1999) and pig (Ricke et al., 1999), indicating that little endothelial cell proliferation occurs during luteal regression. The reduction in VEGF may reduce support for the luteal vasculature, leading to vascular regression. Such a phenomenon has been witnessed in tumors where withdrawal of VEGF will cause regression of the vessels that had built up in that tumor (Benjamin and Keshet, 1997; Baffert et al., 2006), and this phenomenon is a current target for anti-cancer therapeutics (Meadows and Hurwitz, 2012; Frezzetti et al., 2017). However, this has yet to be confirmed in the regressing corpus luteum.

#### 4.1.2 Fibroblast growth factor 2 (FGF2)

Fibroblast growth factor 2, or basic fibroblast growth factor (FGF2) is a heparin binding protein, discovered for its ability to induce proliferation of NIH 3T3 fibroblast cell lines (Gospodarowicz, 1974). FGF2 is a prominent angiogenic factor, potentially even more so than VEGF, and it is necessary for luteal formation, as blockade of FGF2 at the time of ovulation results in corpora lutea that have malformed blood vessels and lower progesterone (Robinson et al., 2007; Yamashita et al., 2008). Immunohistochemical studies reveal FGF2 localization in endothelial cells and steroidogenic cells of the corpus luteum (Asakai et al., 1993; Alan and Kulak, 2021).

Unlike VEGF, FGF2 expression is increased during luteal regression (Neuvians et al., 2004a; Neuvians et al., 2004b; Zalman et al., 2012; Monaco et al., 2023). These findings indicate that an elevation in luteal FGF2 is not sufficient to prevent the loss of endothelial cells and capillaries that occurs during early luteal regression, nor does FGF2 cotreatment affect progesterone levels of cows treated with PGF2α *in vivo* (Piotrowska*-*Tomala et al., 2021). An explanation for this phenomenon could be that the increased FGF2 transcription is a compensatory effect and slows down the luteolytic process (Neuvians et al., 2004b). Another explanation is that FGF2 acts on stromal or fibrotic components of the corpus luteum. In support of this idea, using smooth muscle actin as a marker, Reynolds and Redmer observed that fibroblast number was elevated in the corpus luteum during the late luteal phase of sheep, and collagen production was also elevated following PGF2α treatment (Reynolds and Redmer, 1999; Vonnahme et al., 2006). A recent study showed that FGF2 induced both proliferation and collagen production of bovine luteal fibroblasts (Monaco et al., 2023). Furthermore, *in vitro* studies reveal that the presence of type 1 collagen causes endothelial cells to become more sensitive to TGFB1 (Maroni and Davis, 2011). Therefore, FGF2 in the regressing corpus luteum may be acting as a luteal pro-fibrotic factor that contributes to sensitizing endothelial cells to luteolytic ligands.

#### 4.1.3 Angiopoietins 1 & 2 (ANGPT1/2)

Angiopoietins (ANGPTs) are growth factors that regulate angiogenesis through binding to tyrosine kinase receptors Tie1 and Tie2. ANGPT1 is known to be secreted by pericytes and smooth muscle cells adjacent to endothelial cells, and ANGPT2 is believed to be exclusive to endothelial cells (Eklund and Saharinen, 2013). However, *in situ* hybridization and immunohistochemistry of the human corpus luteum revealed ANGPT 1 and 2 distribution in both steroidogenic and endothelial cells (Wulff et al., 2000; Sugino et al., 2005), and a similar result was revealed in the buffalo corpus luteum (Mishra et al., 2016). ANGPT1 binds to Tie2 on endothelial cells to promote angiogenesis by inducing proliferation and migration. In contrast, ANGPT2 acts as a Tie2 receptor antagonist to ANGPT1 and plays a role in vessel stabilization rather than new vessel formation (Maisonpierre et al., 1997). The ANGPT2/ANGPT1 ratio was reported to be elevated in regressing corpora lutea, suggesting that angiogenesis does not occur during luteal regression (Goede et al., 1998). Expression of *ANGPT1* transcripts in corpora lutea may be species-dependent, as it was highest at mid-luteal phase in bubaline (Mishra et al., 2016) and bovine (Tanaka et al., 2004) corpora lutea, and in corpora lutea of pregnancy in human (Sugino et al., 2005), but highest at late cycle in rhesus monkey corpora lutea (Hazzard et al., 2000). *ANGPT2* expression was lowest at midluteal phase in human corpora lutea and decreased in corpora lutea of pregnancy, when the corpus luteum was fully functional (Sugino et al., 2005), whereas it was highest in the late luteal phase in the rhesus monkey (Hazzard et al., 2000) and bubaline corpus luteum (Mishra et al., 2016). In the bovine corpus luteum, no changes in luteal *ANGPT2* mRNA were detected throughout the luteal phase but *ANGPT2* mRNA was transiently elevated 2 h post-PGF2α injection, and then decreased after 4 h of PGF2α treatment (Tanaka et al., 2004). Although there are no reports of the effects of ANGPT2 on luteal endothelial cells, ANGPT2 has been shown to sensitize endothelial cells to the pro-apoptotic effects of TNF (Fiedler et al., 2006). Tanaka et al. found that ANGPT2 decreased progesterone secretion of bovine corpora lutea at 4–8 h of treatment at higher concentrations (100 ng/mL) in an *in vitro* microdialysis system (Tanaka et al., 2004). This is inconsistent with what was reported in the buffalo, in which ANGPT1 and 2 increased progesterone production in cultured bubaline luteal cells after 24 h of treatment at 100 ng/mL (Mishra et al., 2016). Because ANGPTs are known to act primarily on endothelial cells (Eklund and Saharinen, 2013), more studies using primary luteal endothelial cells are needed to elucidate their role in angioregression.

### 4.2 Anti angiogenic factors

#### 4.2.1 Thrombospondin 1 (THBS1)

Thrombospondins are a family of calcium-binding glycoproteins that influence a variety of functions, including angiogenesis, vessel biology, and wound healing. They are known to regulate several factors such as AT2, TGFB1, and even matrix metalloproteinases (Adams and Lawler, 2011), but the well-established function of thrombospondins 1 and 2 is dysregulation of angiogenesis (Armstrong and Bornstein, 2003). The role of thrombospondins in the corpus luteum has been reviewed in detail (Farberov et al., 2019). Localization of THBS1 was identified in endothelial cells and steroidogenic cells in the corpus luteum (Berisha et al., 2016b), and *THBS1* expression is increased in the regressing corpus luteum and is reduced in the corpus luteum of pregnancy (Mondal et al., 2011; Zalman et al., 2012; Romero et al., 2013; Farberov and Meidan, 2016). Treatment of luteal tissue slices with interferon tau, the maternal signal for luteal rescue in bovines, decreased *THBS1* mRNA and protein (Basavaraja et al., 2017). It is believed that THBS1 exerts anti-angiogenic effects on the regressing corpus luteum, as it has been demonstrated to decrease numbers of bovine and rabbit luteal endothelial cells in culture (Bagavandoss and Wilks, 1990; Farberov and Meidan, 2016). Thrombospondin 1 may act in part through TGFB1 signaling, as THBS1-treated luteal cells exhibited increased downstream TGFB1 signaling, which was decreased with THBS1 knockdown; however, inhibition of TGFB1 signaling did not ameliorate THBS1-induced endothelial cell death (Farberov and Meidan, 2016). Therefore, other signaling factors must come into play. For instance, a TGFB superfamily member, Nodal, which is increased in hypoxic conditions, elevated THBS1 and CD36 and downregulated CD31 (an endothelial cell marker) in equine luteal explants (Walewska et al., 2019). Furthermore, despite its elevation in luteolysis, THBS1 has been shown to be upregulated after ovulation and induced migration of monkey ovarian endothelial cells (Bender et al., 2019). Therefore, the action of THBS1 must depend on multiple signaling pathways and the presence of other factors in the corpus luteum, as the pro or anti-angiogenic effect of THBS1 is dependent on the target receptor. Thrombospondin 1 acts as an anti-angiogenic factor when bound to CD36 (Dawson et al., 1997), and as a pro-angiogenic factor when bound to the low density lipoprotein receptor-related protein-1 (LRP1) receptor (Orr et al., 2003). CD36 was elevated in PGF2α-induced luteolysis of the bovine corpus luteum (Berisha et al., 2016b). However, it has been shown in the ovary that THBS1 can inhibit VEGF through LRP1 binding (Greenaway et al., 2007). THBS1 has also been demonstrated to sequester FGF2 by binding to its heparin-binding site (Farberov et al., 2019). The ability of THBS1 to bind to many targets including angiogenic factors and cytokines such as TGFB1, makes it an ideal candidate for further investigation of factors that can influence cytokine levels and angioregression in the corpus luteum.

## 5 Effects of cytokines on endothelial cells

### 5.1 Tumor necrosis factor α (TNF)

In most cells, TNF binding to its receptor (TNFR1) results in inflammatory responses and/or programmed cell death. TNF protein is elevated in the pseudopregnant mouse corpus luteum following treatment with a luteolytic dose of PGF2α (Henkes et al., 2008). In the bovine, mRNA for TNF is upregulated early in PGF2α induced luteolysis (Talbott et al., 2017) and in the natural cycle (Friedman et al., 2000; Sakumoto et al., 2000). TNF has been shown to be localized to immune cells and faintly in steroidogenic cells in the bovine corpus luteum (Sakumoto et al., 2011); however, others have found TNF to be localized in the vascular immune cells of the porcine corpus luteum (Hehnke-Vagnoni et al., 1995). TNF affects steroidogenesis directly by decreasing LH-stimulated progesterone production in luteinized murine granulosa cells (Adashi et al., 1990) and in porcine (Pitzel et al., 1993) and bovine (Benyo and Pate, 1992; Sakumoto et al., 2000) luteal cells. Furthermore, TNF can induce production of PGF2α, thus creating a positive feedback loop within the regressing corpus luteum (Sakumoto et al., 2000).

Luteal endothelial cells are sensitive to inflammatory cytokines, as TNF induces apoptosis of luteal endothelial cells (Friedman et al., 2000; Pru et al., 2003; Cherry et al., 2008; Henkes et al., 2008). Endothelial cells are a rich source of acid sphingomyelinase (ASMase), a common mediator of cytokine signaling. A report by Henkes et al. shows that TNF, but not PGF2α, activated ASMase and cell death in murine ovarian endothelial cells, a response that was not present in endothelial cells lacking ASMase (Henkes et al., 2008). *In vivo* studies revealed that mice treated with Etanercept, a TNF receptor inhibitor, were resistant to PGF2α-induced luteal regression. Additionally, mice lacking ASMase were protected from PGF2α-induced luteal regression. There was no gross evidence of PGF2α-induced disruption of the corpus luteum in the ASMase deficient mice, findings supported by the maintenance of progesterone levels (Henkes et al., 2008). These results suggest that TNF-mediated activation of ASMase serves a pivotal role in altering the luteal vasculature during PGF2α-induced luteal regression. The absence of direct cytotoxic effects of PGF2α on isolated luteal endothelial cells argues that luteal regression in response to PGF2α requires cytokines like TNF to disrupt vascular integrity. *In vitro* studies with bovine luteal endothelial cells show that TNF also induces apoptosis by inducing ASMase, production of ceramide, and activation of Jun-N-terminal Kinase (JNK) MAPK signaling (Pru et al., 2003). TNF-induced endothelial cell death may increase vascular permeability, allowing for increased infiltration of immune cells, which release more TNF, thereby exacerbating angioregression. However, further studies need to be performed to determine whether TNF affects luteal vascular permeability *in vivo*.

### 5.2 Interleukin 1 β (IL1B)

Interleukin 1 β (IL1B) is a pro-inflammatory cytokine that, like TNF, is upregulated early in luteal regression (Talbott et al., 2017). Also like TNF, IL1B is produced in immune cells, based on previous studies (Lopez-Castejon and Brough, 2011), although some studies indicate that fibroblasts may be able to secrete inflammatory cytokines like IL1B (Kobayashi and Terao, 1997; Bartok and Firestein, 2010), and a transcriptomic study identified the presence of *IL1B* mRNA in small steroidogenic cells of the bovine corpus luteum (Baddela et al., 2018). Although the exact localization of IL1B has not been determined in the corpus luteum, flow cytometry of the primate corpus luteum indicated that IL1B is produced by CD11b^+^ cells in the late luteal phase (Bishop et al., 2017). Unlike TNF, the role of IL1B has not been investigated in luteal endothelial cells; however, in the corpus luteum, IL1B has been demonstrated to impair steroidogenesis and increase cyclooxygenase and subsequent PGF2α production in cultured rat ovarian tissue (Estevez et al., 2003), indicating cytokines like IL1B can potentially contribute to a positive-feedback loop to enhance PGF2α production and action.

In studies of human umbilical vein endothelial cells (HUVECs), IL1B induces matrix metalloprotease 9 and decreases levels of tissue inhibitor of matrix metalloprotease 1, suggesting that IL1B induces a net breakdown of matrix (Qin et al., 2012). In the same study, it was demonstrated that IL1B also increased endothelial cell permeability and altered expression of membrane cadherins and claudins, which could implicate increased immune cell recruitment (Qin et al., 2012). In agreement with this possibility, both TNF and IL1B increased neutrophil recruitment to HUVECs. Furthermore, the effect of IL1B was increased slightly in conditions of shear stress (Sheikh et al., 2005). A potential mechanism could be through the secretion of chemokines, which attract immune cells like neutrophils. For instance, IL1B increased secretion of IL8, a chemokine known to recruit neutrophils, in immortalized human microvascular endothelial cells (Bourdiec et al., 2013). Additionally, the receptor for IL1B, IL1R, has been implicated in shear-stressed induced EndMT, where endothelial cells transdifferentiate into myofibroblast-like cells (Kidder et al., 2023). Therefore, IL1B action in angioregression may be beyond generalized inflammation, and may even induce further inflammation through recruitment of immune cells and increased production of PGF2α.

### 5.3 Transforming growth factor β1 (TGFB1)

Similar to TNF and IL1B, TGFB1 is increased very early during luteal regression (Hou et al., 2008; Talbott et al., 2017). TGFB1 is a ubiquitously expressed cytokine that binds to tyrosine kinase TGFB receptors (TGFBRs) and activates a family of transcription factors called mothers against decapentaplegic, commonly known as SMADs, by phosphorylation (Branton and Kopp, 1999; Tzavlaki and Moustakas, 2020). One of the classic functions of TGFB1 is activating fibroblasts into myofibroblasts, in which they gain more actin fibers and produce more collagen (Vallée and Lecarpentier, 2019). Maroni and Davis demonstrated that TGFB1 induces collagen and laminin production by bovine luteal fibroblasts *in vitro* (Maroni and Davis, 2012). In luteal endothelial cells, TGFB1 decreased DNA incorporation, cell migration, and endothelial sprouting (Maroni and Davis, 2011). In that same study, TGFB1 increased caspase-3/7 activity of bovine luteal endothelial cells, disrupted the distribution of VE-cadherin, and increased endothelial cell permeability (Maroni and Davis, 2011). These findings demonstrate that TGFB1 has a direct effect on luteal angioregression by elimination of endothelial cells, increased vascular permeability, and fibrosis of the vasculature.

## 6 Summary and future directions

Although the exact mechanism of vascular regression of the corpus luteum remains unclear, it is known that blood flow transiently increases then is impaired. The early increase in blood flow may be from the release of vasodilators like NO during luteal regression. Based on observations in other tissues, shear stress may alter endothelial cell morphology and consequent function (Malek and Izumo, 1996; Watts, 2007). As mentioned in the review, endothelial cells themselves are also sensitive to growth factors and cytokines released during luteal regression. Increased production of cytokines in the luteal tissue microenvironment during regression may contribute to such changes in morphology as well as death of endothelial cells. The cytokine-mediated events, in conjunction with the loss of growth factors that typically signal endothelial cell survival and proliferation, contribute to angioregression in the corpus luteum. Endothelial cell apoptosis may result in sloughing of cells into capillaries and increased permeability of the vessels.

In this review, we summarized the current knowledge on changes in blood flow, presence of vasoactive factors and inflammatory cytokines, which all may contribute to angioregression of the corpus luteum. However, endothelial cells themselves may also be acting as luteolytic agents. Luteal endothelial cells can produce cytokines such as TNF as well as chemokines like monocyte chemoattractant 1 protein, which attracts immune cells to the site of luteolysis (Liptak et al., 2005; Cherry et al., 2008). Furthermore, *in vivo* studies revealed that PGF2α rapidly induced P-selectin, a leukocyte adhesion molecule, in bovine endothelial cells and recruited polymorphonuclear neutrophils into the corpus luteum (Shirasuna et al., 2012a). Studies in the primate indicate infiltration of macrophages, neutrophils, and natural killer cells in the regressing corpus luteum (Bishop et al., 2017). Endothelial-immune cell interactions may exacerbate luteolysis, as *in vitro* studies revealed that contact co-culture of endothelial cells with peripheral blood mononuclear cells was required to synergistically increase monocyte chemoattractant protein 1 (Liptak et al., 2005). Endothelial cells also express class II major histocompatibility (MHC) proteins, which allow binding and subsequent activation of T lymphocytes (Cannon et al., 2007a). Luteal endothelial cells also express proteins that stabilize MHC II binding to T-cells as well as the costimulatory molecule CD80, which induces T-cell survival and proliferation (Cannon et al., 2006; Cannon et al., 2007b). However, most of the current research focuses on the influence of endothelial cells on immune cells rather than *vice versa*. Studies are needed to determine how immune cell binding to luteal endothelial cells affects vascular permeability, recruitment of other immune cells and viability.

This review has centered on the actions of a number of growth factors and cytokines, but newly developing research implicates additional factors like adipokines and neuropeptides in luteal regression. The potential role of many of these factors is the subject of a recent review (Mlyczyńska et al., 2022). Many of these factors impact metabolic pathways in cells that ultimately control cellular fate. How adipokines and neuropeptides contribute to luteal angioregression is a question requiring further investigation.

It is well established that structural regression of the corpus luteum involves programmed cell death of endothelial cells and steroidogenic cells. However, recent research indicates that apoptosis, autophagic cell death, and necroptosis may all contribute to regression of the bovine corpus luteum (Hojo et al., 2022). It is not known whether and how each of these processes contribute to the demise of endothelial cells. Additionally, non-apoptotic pathways of angioregression may also occur in the regressing corpus luteum, such as a change in endothelial cell phenotype. Senescence of vascular endothelial cells has been associated with vascular dysfunction in variety of disease states by impairing new angiogenesis and endothelial-mediated changes in vascular tone (Shosha et al., 2018; Kiss et al., 2020; Cohen et al., 2021; Bloom et al., 2023a; Bloom et al., 2023b; Han and Kim, 2023). Senescent cells exhibit a pro-inflammatory phenotype (Birch and Gil, 2020); thus, if endothelial cell senescence is present in the regressing corpus luteum, then these senescent endothelial cells may be producing the inflammatory mediators that contribute to angioregression and the viability of steroidogenic cells. Research is needed to determine the phenotypes of luteal endothelial cells before and during luteal regression.

Another possibility is that some endothelial cells are changing phenotype by transdifferentiation or EndMT. This phenomenon has been implicated in vascular dysfunction of various disease states such as cancer, vascular fibrosis, sclerosis, pulmonary hypertension, and atherosclerosis (Zeisberg et al., 2007; Manetti et al., 2017; Alvandi and Bischoff, 2021; Cai et al., 2021; Gorelova et al., 2021). EndMT is known to be initiated by TGFB1, and is exacerbated by TNF in cancer-associated fibroblasts (Yoshimatsu et al., 2020); therefore, it is possible that the increases in content of TNF and TGFB1 during luteal regression can contribute to EndMT. Because luteal endothelial cells exhibit a variety of phenotypes (Spanel-Borowski and van der Bosch, 1990; Spanel-Borowski, 1991; Fenyves et al., 1994; Davis et al., 2003), it is possible that different endothelial cells undergo different forms of regression, for instance cell death compared to senescence or EndMT. Lineage tracing models using a transgenic animal model can be used to study EndMT *in vivo* (Sánchez-Duffhues et al., 2018), but has yet to be done in the corpus luteum to track whether EndMT occurs or when it occurs in the regression process. It is possible that EndMT may appear later in regression, when fibrosis occurs, following inflammation.

Furthermore, many of these previous studies are done in cultured endothelial cells from whole digested luteal tissue. Thus, arteriolar, venular, and microvascular endothelial cells are all considered to be the same. However, studies in other tissues indicate that the type of vessel from which the endothelial cell belonged can have an effect of its responsiveness to certain environmental stressors. For instance, Polk et al. demonstrated that human dermal microvascular cells slightly oriented themselves in response to shear stress, and Reinitz et al. demonstrated that human brain microvascular endothelial cells did not change shape as HUVECs did in response to shear stress (Reinitz et al., 2015; Polk et al., 2022). It was also demonstrated that rat endothelial cells from different vessels respond differently to vasoactive factors, such as acetylcholine-induced relaxation (Abukabda et al., 2017). In that study, it was identified that titanium dioxide nanoparticles impaired acetylcholine-induced relaxation in endothelial cells from the aorta, third-, and fourth-order mesenteric arteries, whereas it had no effect in endothelial cells from the femoral artery (Abukabda et al., 2017). Additionally, human dermal microvascular endothelial cells exhibited less damage in response to *Candida albicans* compared to HUVECs but more damage in response to *Staphylococcus aureus* (Seidl et al., 2012). The human microvascular endothelial cells also released less IL-8 compared to HUVECs in response to either pathogen (Seidl et al., 2012). Because of such differences between macro- and microvascular endothelial cell responses to pathogens and shear stress, it is important to decipher the macro- and microvascular responses separately when studying luteal angioregression. Various changes in the vasculature and causes of endothelial cell death have been identified in the corpus luteum (summarized in Table 1). However, a unifying mechanism of vascular regression has yet to be established. The current knowledge revolves around changes in blood flow and secreted factors that may affect endothelial cell viability; yet, neither the exact transcriptome or proteome of regressing vascular endothelial cells nor their varying phenotypes has yet to be studied. Single-cell proteomic or transcriptomic studies conducted during luteal regression could provide new insight on how changes in the luteal microenvironment in response to PGF2α lead to angioregression.

Furthermore, the fibrosis of various luteal vessels has yet to be explored. Studies show that while the microvasculature is disappearing the walls of larger vessels become thicker during luteal regression (Bauer et al., 2003). The presence of myofibroblast-like cells in the regressing corpus luteum (Nio-Kobayashi et al., 2016) may contribute to a fibrotic response. As reviewed above, many endothelial-reactive factors may contribute to fibrosis. Fibroblast activation by TGFB1 and FGF2 (Monaco et al., 2023) and deposition of matrix may be a major contributor to this process. EDN1 has been implicated in pulmonary fibrosis and TNF-induced EndMT in cardiac fibrosis (Swigris and Brown, 2010; Hu et al., 2023). Thrombospondins may also contribute to vascular fibrosis through TGFB1 (Sweetwyne and Murphy-Ullrich, 2012). Given that the end stage of luteal regression is a fibrotic corpus albicans, more studies are needed to elucidate the mechanism of luteal vascular regression beyond blood flow and apoptotic cell death of endothelial cells.
